# The complete mitochondrial genome of the *Rana sangzhiensis* Shen and its phylogenetic analyses

**DOI:** 10.1080/23802359.2020.1866454

**Published:** 2021-02-03

**Authors:** Gang Xiong, Hui Jiang, Xiao-Qing Wang, Li Kang, Li Liu, Xian-Wen Zhou, Pei Wang, Tao Yang

**Affiliations:** a Hunan Biological and Electromechanical Polytechnic, Changsha, Hunan, China; b College of Animal Science and Technology, Hunan Agriculture University, Changsha, Hunan, China; c Hunan Fisheries Science Institute, Changsha, Hunan, China

**Keywords:** Mitochondrial DNA, *Rana sangzhiensis* Shen, phylogenetic analysis

## Abstract

The mitochondrial genome of one *Rana pseudo-rana* species *Rana sangzhiensis* Shen was sequenced and annotated. The mitogenome is 19,207 bp in length, containing 37 typical genes. The A + T content of the whole mitogenome is 56.6%. All of the protein-condoning genes (PCGs) started with ATG and stopped with TGA. The tRNA-Pro, tRNA-Gln, tRNA-Ala, tRNA-Asn, tRNA-Cys, tRNA-Tyr, tRNA-Ser, tRNA-Glu, andND5 are located in the circular mitochondrial L chain. The phylogeny tree is monophyletic among 14 related *Rana* species. The *R. sangzhiensis* Shen cluster was more closely related to *R. amurensis* Boulenger and *R. kunyuensis* Lu, Y.-Y., and P.-P. Li. This mitochondrial genome can be used for further analyses of *Ranidae* mitochondrial comparative genomics to improve the understanding of diverse *Ranidae* species.

The *Rana sangzhiensis* as a kind of small and medium-sized frog belonging to *Rana pseudo-rana*, *Ranidae*. The *R. sangzhiensis* Shen is distributed in Chenzhou, Yongzhou, Hengyang, Loudi, Huaihua and Xiangxi City, Hunan province, China. The *R. sangzhiensis* Shen generally lives in stream migration water and the surrounding grass with the mountain altitude of 800–1000 meters above sea level, and comes out to hunt for food in the day, and in the night only to mate in the breeding season (Xiang-Rong et al. 2008).

A male adult of *R. sangzhiensis* Shen was collected from Xingning Town (Zixing, Chenzhou City, Hunan Province, China) (113°43.0′E, 27°99.9′N), in October 2019. The specimens (No. Zg-zx) were stored at −80 °C in our laboratory (College of Animal Science and Technology, Hunan Agriculture University). Total genomic DNA of the *R. sangzhiensis* Shen was extracted from fertilized eggs using the Column Animal DNA out (Beijing Tiandz, Beijin, China) following the kit’s instructions. Then, the mitochondrial genome (mitogenome) of *R. sangzhiensis* Shen was sequenced by Illumina sequencing platform (Wuhan Biowefind biotechnology Co., Ltd, Wuhan, China). The reads were assembled and annotated using Generous Prime (v2019.1.3.). All tRNA genes were identified by ARWEN v1.2 (Laslett and Canbäck 2008). The annotated sequences of mitogenome were submitted to GenBank with the accession number MT782121. All protein-condoning genes (PCGs) were aligned using MAFFT algorithm in the TranslatorX (Katoh et al. 2019), and then poorly aligned results were removed by Gblocks 9.1 b (Castresana 2000; Abascal et al. 2010).

Mitogenome of *R. sangzhiensis* Shen has 19,207 bp in length, including 37 typical genes (13 PCGs, 22 tRNA genes and 2 rRNA genes). The overall nucleotide composition of the mitogenome is 27.3% for A, 28.5% for C, 14.7% for G and 29.3% for T. The A + T content of genes is 56.6%. All of PCGs started with ATG and stopped with TGA. The tRNA-Pro, tRNA-Gln, tRNA-Ala, tRNA-Asn, tRNA-Cys, tRNA-Tyr, tRNA-Ser, tRNA-Glu and ND5 are located in the circular mitochondrial L chain. The length of 22 tRNA is range from 67 bp (tRNA-Ser) to 74 bp (tRNA-Asn). Genes of 16S rRNA and 12S rRNA are 1576 bp and 930 bp, respectively. To validate the phylogenetic position of *R. sangzhiensis* Shen, we perform multiple sequence alignment by MEGA 6.0 (Tamura et al. 2013) and construct a maximum likelihood tree based on the complete mitochondrial genome sequences of 16 species in *Ranidae*. The phylogenetic tree (Figure 1) revealed that 14 *Rana* species constituted five subclades, the *R. sangzhiensis* Shen clustered in one clade, the *R. sangzhiensis* Shen cluster was more closely related to *R. amurensis* Boulenger and *R. kunyuensis* Lu, Y.-Y. and P.-P. Li.

**Figure 1.**
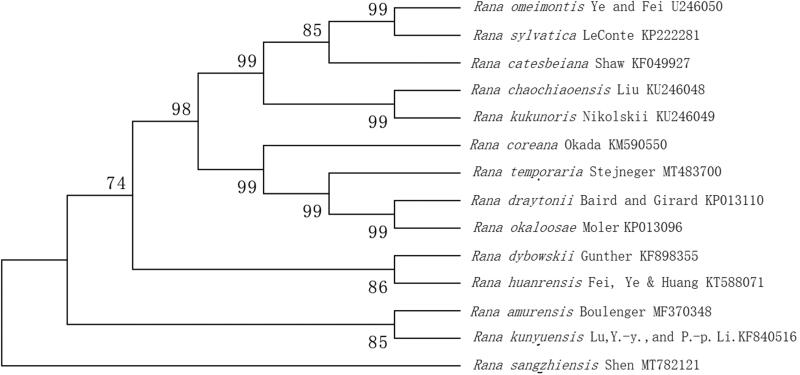
Phylogenetic analysis infers the evolutionary relationship of R. sangzhiensis Shen. The tree was constructed based on maximum likelihood (ML) tree method using Mega 6.0. The GenBank accession number for each species is indicated after the scientific name.

## Data Availability

The genome sequence data that support the findings of this study are openly available in GenBank of NCBI at (https://www.ncbi.nlm.nih.gov/) under the accession no. MT782121. The associated BioProject, SRA, and Bio-Sample numbers are 20TD07, PRJNA678467, and Zg-zx respectively.

## References

[CIT0001] Abascal F, Zardoya R, Telford MJ. 2010. TranslatorX: multiple alignment of nucleotide sequences guided by amino acid translations. Nucleic Acids Res. 38(Web Server issue):W7–W13. 20435676 10.1093/nar/gkq291 PMC2896173

[CIT0002] Castresana J. 2000. Selection of conserved blocks from multiple alignments for their use in phylogenetic analysis. Mol Biol Evol. 17(4):540–552. 10742046 10.1093/oxfordjournals.molbev.a026334

[CIT0003] Xiang-Rong He, Xiao-Qing Wang, Wu-Jiang Fan, Xian-Wen Zhou. 2008. Analysis of biological characters and nutritional components of *Rana sangzhiensis*. J Hunan Agric Univ (Nat Sci). 8.34(4):482–484.

[CIT0004] Katoh K, Rozewicki J, Yamada KD. 2019. MAFFT online service: multiple sequence alignment, interactive sequence choice and visualization. Brief Bioinform. 20(4):1160–1166. 28968734 10.1093/bib/bbx108 PMC6781576

[CIT0005] Laslett D, Canbäck B. 2008. ARWEN: a program to detect tRNA genes in metazoan mitochondrial nucleotide sequences. Bioinformatics. 24(2):172–175. 18033792 10.1093/bioinformatics/btm573

[CIT0006] Tamura K, Stecher G, Peterson D, Filipski A, Kumar S. 2013. MEGA6: molecular evolutionary genetics analysis version 6.0. Mol Biol Evol. 30(12):2725–2729. 24132122 10.1093/molbev/mst197 PMC3840312

